# Interoceptive sensibility and body satisfaction in pregnant and non-pregnant women with and without children

**DOI:** 10.1038/s41598-022-20181-z

**Published:** 2022-09-27

**Authors:** Anna Crossland, Elizabeth Kirk, Catherine Preston

**Affiliations:** 1 University of York, York, England, UK; 2 Anglia Ruskin University, Cambridge, England, UK

**Keywords:** Human behaviour, Risk factors

## Abstract

Pregnancy is a time of great physical and psychological change. As well as prominent changes in the external appearance of the body, such as the baby bump, there are also substantial changes taking place within the body. Our awareness of, and attention towards, internal bodily signals (interoception) is thought to have a direct impact on how we feel about our bodies. Therefore, understanding how our experience of these interoceptive signals might change during pregnancy may have important implications for maternal wellbeing. This study examined body satisfaction and interoceptive sensibility (subjective experience of interoception) in pregnant and non-pregnant women with and without children. Feelings towards pregnancy-specific changes in body satisfaction and interoceptive sensibility were also examined in women in their first pregnancy (primigravida) and subsequent pregnancies (multigravida). It was found that pregnancy did not directly impact levels of body satisfaction, instead pregnant and non-pregnant women with children reported less satisfaction with their bodies compared to those without children. Primigravida women were more satisfied with the appearance of pregnancy specific bodily changes compared to multigravida women. Interestingly, these differences in body satisfaction in those with children (pregnant and non-pregnant) were mediated by the extent to which women trusted their bodies (measure of interoceptive sensibility). All other pregnancy related changes in interoceptive sensibility and body satisfaction were either non-significant or had small effect sizes. These results may suggest body trust as an important factor to support during the transition to parenthood in order to improve body satisfaction in mothers.

## Introduction

During pregnancy, an expectant mother experiences vast physiological and psychological adjustments in a relatively short period of time (approximately 40 weeks). Many of these changes are externally visible, such as the abdominal area protruding increasingly through the duration of the pregnancy (baby bump), weight gain^1^, changes in gait and foot width^2^, and appearance of the skin and hair^3^. Much of the research to date examining women’s experiences of the body during pregnancy focuses on such external bodily changes, and how that makes her feel about herself and the fetus. For example, how women feel about and adapt to external bodily changes during pregnancy is thought to impact on mental health^4,5^ and physical health behaviours^6^. In addition, pregnancy body satisfaction is found to influence infant wellbeing through the development of antenatal attachment^7^ and breastfeeding intentions and duration^8^, which are important for infant development^9,10^.

There is noticeably less literature investigating the changes in internal bodily signals through pregnancy, and how such changes influence psychological wellbeing. The awareness of internal bodily signals such as heart rate, hunger and thirst is widely referred to as interoception^11^, which has a physiological basis correlating directly with activation in the anterior insular cortex^12^. There are three distinct mechanisms which are considered to form interoception^13^; interoceptive accuracy (or sensitivity), interoceptive sensibility and interoceptive awareness. Interoceptive *accuracy* refers to how accurate an individual is at detecting and interpreting interoceptive signals when compared with objective measures^14^. Conversely, interoceptive *sensibility* refers to the subjective experience of interoception, or how interoceptive signals are experienced irrespective of their objective reality^15^, primarily measured with self-report questionnaires. Finally, interoceptive *awareness* refers to the correspondence between objective interoceptive accuracy and subjective reports; metacognitive awareness of one’s own interoceptive accuracy^13^. Dysfunction in interoception is increasingly recognised as an important component of mental ill-health, including eating disorders^16–18^, depression and anxiety^19,20^ and schizophrenia^21^. Recent research suggests this is also the case during pregnancy^7,22^.

During pregnancy women may experience many visceral changes, such as increased hunger and thirst due to greater required energy intake^23^ and the heightened importance of hydration^24^. There are also changes in perception and experience of fatigue during the three trimesters^25^ and increased cardiac output^26^ resulting in increased body temperature^27^. Cardiac output, and notably detection of strength and speed of heartbeat are considered key markers of interoceptive accuracy^13^. Moreover, there are physiological experiences unique to the gestational period, such as pregnancy related pain^28^.

Awareness and understanding of these bodily signals may be important during pregnancy for the safety of the mother and fetus. There is an increased emphasis on listening to signals from within the body, such as fetal movements^29^, with advice from healthcare professionals to recognise and monitor fetal movements due to the importance for detecting potentially life threatening fetal anomalies^30^. Although movements of the fetus do not originate from the women's own body so may not constitute interoception in themselves, detecting such movements is likely to change the way women attend to their body. Additionally, fetal movements are also likely to impact interoceptive signals by additional noise or by directly affecting visceral organs such as the bladder. The way women attend to their body is also likely to change during pregnancy, with women listening to and trusting their body more when pregnant^7^. Feelings of trusting the body are thought to be important during pregnancy, specifically trusting the body to support the safe development of the fetus, which women have reported is a critical part of late pregnancy^31^, and trusting that the body will undertake the necessary requirements during labour^32^, such as knowing when to push.

The other aspect of bodily experience to be examined in the current study relates to how women feel about their external appearance (body satisfaction). Many studies examining body satisfaction in pregnant samples use scales which ask about satisfaction with different aspects of the body, such as the Body Cathexis Scale^33^, however, such scales have not been validated for pregnancy and do not dissociate between different aspects of body satisfaction. For this study we will use a body satisfaction scale that has been developed explicitly for pregnancy body satisfaction, the Body Understanding Measure of Pregnancy scale (BUMPs)^7^. This measure captures three separate aspects of pregnancy body satisfaction: Appearance, measuring satisfaction with the appearance of being pregnant; Weight, measuring concerns about pregnancy weight gain; and Physical, measuring frustrations with the physical burdens of pregnancy. Previous studies have suggested that changes in body satisfaction through pregnancy vary depending on the construct being measured. For example, women report feeling less fat during later stages of pregnancy, whilst other aspects are more stable^34^. Considering that perceived fatness is contrary to social ideals, and that women are more likely to look distinctly pregnant (opposed to overweight) the closer they are to birth, we predict that appearance dissatisfaction and weight concerns would decrease with gestational age. Conversely, due to the increasing size of the baby bump we would predict that frustrations with physical burdens of pregnancy would be higher for women further through pregnancy.

Because of the physical nature of pregnancy and birth, having already experienced this may impact on interoceptive and exteroceptive bodily experience. Multigravida women (women who have been pregnant before) report increased fatigue^35^ and lower self ratings of health^36^ than primigravida women (women in their first pregnancy). Primigravida women are also found to report lower levels of attention regulation (measure of interoceptive sensibility), which suggests that they are less able to sustain and control attention to their body than multigravida women, and this is associated with higher levels of anxiety^22^. In terms of exteroceptive body appearance, multigravidae women have been found to feel more negatively about their body than primigravidae^31^. For first time mothers, pregnancy involves many previously unexperienced bodily changes, and adjustments in awareness of her own physical state. Multigravida women, on the other hand, have already undergone perinatal bodily changes, and thus may know what changes to expect in their body^37^. Additionally perception of, and satisfaction with, bodily changes may depend on a comparison with the pre-pregnant self^37^. For primigravidae the pre-pregnant self could be very different to multigravidae’s pre-pregnant self, whose bodies have already been changed by previous pregnancies^38^ and may have experienced permanent changes to body shape and function^39,40^. Furthermore, non-pregnant women with children have been found to have a more positive relationship with food^41^, but less positive attitudes about bodily appearance^42^, suggesting that being a mother may have an impact on our experience of the body even outside of pregnancy.

The current study aimed to assess changes in interoceptive sensibility and body satisfaction during pregnancy in primigravidae and multigravidae, as well as by comparing to non-pregnant women with and without children. Interoceptive sensibility will be assessed using the Multidimensional Assessment of Interoceptive Awareness (MAIA)^43^. The MAIA consists of eight subscales capturing different constructs of interoceptive sensibility, including noticing and listening to signals from the body, not worrying about and not distracting from internal signals, body trusting, attention regulation, self regulation and emotional awareness. A recent study has suggested that many of these constructs change during pregnancy and into the postpartum period^44^.

It is predicted that due to increased emphasis on bodily signals throughout pregnancy, pregnant women will report that they notice changes in their body, listen more to their body and trust their body more compared to non-pregnant women. Due to the increased importance of attending to the body, we also anticipate pregnant women will demonstrate less interoceptive avoidance and greater worrying, particularly amongst primigravida. In line with previous findings^44^, we also predict that primigravida will report less attention regulation compared to multigravida women. Evidence suggesting that pain and interoceptive sensibility changes between early and late pregnancy^44–47^ leads to the prediction that there will be differences in the aforementioned constructs of interoceptive sensibility between women across gestation, specifically women will notice, listen and worry about signals more and distract less from internal signals (i.e. pain and discomfort) the closer they get to birth. We will also examine potential differences in the remaining interoceptive sensibility constructs (self regulation and emotional awareness) due to recent research suggesting these are greater during pregnancy compared to postpartum^44^. We also predict that permanent bodily changes from previous pregnancy/ies and birth will drive body satisfaction. Thus, women with children (pregnant and non-pregnant) will have lower body satisfaction compared to women in their first pregnancy and those without children.

A single coherent representation of the bodily self comprises both interoceptive and exteroceptive signals from the body, the latter of which includes body satisfaction^48^. This inextricable relationship may suggest that changes in one aspect of the body can impact on the other. For example, those with poor interoceptive sensibility are also thought to have more negative experiences of their own body, and this has been found to be specifically linked with pregnancy body dissatisfaction^7^. Indeed, this relationship may be particularly important when pregnant due to the dramatic physiological changes occurring at this time. Therefore, it is crucial to understand if and how any interoceptive sensibility changes during pregnancy relate to exteroceptive bodily experience. Examining these aspects during pregnancy not only tells us something specifically about the prenatal bodily experience, but it may also help us better understand the mechanistic relationship between interoceptive and exteroceptive bodily signals and wellbeing more generally.

Therefore, we also seek to examine how the link between interoception and external experience of the body (body satisfaction) might change as a result of pregnancy. This will be examined relative to pregnancy specific body satisfaction, and how this compares to non-pregnant women, considering body satisfaction more generally. It is anticipated that pregnancy induced changes in interoceptive signals, which are thought to be primarily driven by supporting the growing fetus, along with changes about how we feel about these signals in pregnancy, may weaken the relationship between body satisfaction and interoception and as such being pregnant will remain the strongest predictor for body satisfaction over and above interoceptive sensibility. Finally, due to the body trusting subscale on the MAIA being found to correlate most strongly with body satisfaction in both pregnant^7^ and non-pregnant^49^ populations this subscale is predicted to play an important role in both general and pregnancy specific body satisfaction.

## Methods

### Participants and procedure

The study was approved by the university departmental ethics committee at The University of York, and all procedures were performed in accordance with relevant named guidelines and regulations. Pregnant and non-pregnant women responded to separate advertisements to complete online surveys about bodily experience, and whether they had children, hosted on Qualtrics (Provo, UT). The advertisements were distributed via social media sites (Twitter, Facebook), university staff newsletters, parenting websites, groups and classes, a local maternity ward and a maternity retailer. Informed consent was gained from all participants before commencing the study. The sample of 259 pregnant women was selected from a larger sample^7^ that consisted of women who had completed all the relevant measures (see below). The non-pregnant women were recruited to complete a separate survey to provide a control sample. Pregnant respondents were also categorised as being pregnant with their first baby (primiparous; n = 142) or a subsequent pregnancy (multiparous; n = 117), see Table 1 for more demographic detail of the pregnant sample.

**Table 1:** Details of pregnancy in pregnant participants.

	%
**Gravidity**	
Primiparous	55 (*n* = 142)
Multiparous	45 (*n* = 117)
**Trimesters**	
1	8 (*n* = 22)
2	38 (*n* = 98)
3	54 (*n* = 139)
Multiple births	1 (*n* = 3)

For the non-pregnant sample, a total of 705 women started the survey; respondents were excluded if they were currently pregnant (n = 41), under the age of 18 (n = 1), over the age of 41 (n = 131), had a baby within the last 12 months (n = 120) or had incomplete responses (n = 171), leaving a final sample of 241 women who were not currently pregnant at the time of the research. Women over the age of 41 were excluded in order to make the age range equivalent to the pregnant sample. Literature suggests there may be some residual effects of pregnancy associated with interoception such as changes to pain threshold and to levels of fatigue, that continue for several months after birth^50–52^. To eliminate possible confounds women in the postnatal period (up to 12 months postpartum) were also excluded. This non-pregnant subsample included women who reported having had children (n = 133) and those who reported not to have children (nulliparous; n = 108). The majority of respondents from all groups were white, in a relationship and had an undergraduate degree or higher (Table 2).

**Table 2:** Demographic information for the whole sample.

	Pregnant	Control
N	259	241
Age: mean (SD) [range]	32.2 (4.49) [20–41]	32.7 (5.44) [19–41]
Relationship status: %in a relationship	98.9	86
Race: %white/British	90	92
Education: %undergraduate degree or higher	83	77

### Measures

#### Body cathexis scale^33^ (BCS)

The BCS is a self-report questionnaire that measures satisfaction with the body. For the current study an adapted version of the BCS was used consisting of 43-items^7^. Each item relates to satisfaction with a part of the body or bodily functions, for instance: hands, body build, eyes, health, and weight. Responses are scored on a 5-point Likert scale, ranging from 1 (very dissatisfied) to 5 (very satisfied) and scored by summing all items. Higher scores indicate higher levels of body satisfaction. The scale has good test–retest reliability (0.89) and internal validity^53^ (α = 0.78–0.87). In the current study the internal validity is high for both pregnant (α = 0.92) and non-pregnant (α = 0.93) samples.

#### Multi-dimensional assessment of interoceptive awareness^43^ (MAIA)

The MAIA is a 32-item self-report questionnaire measuring independent constructs of interoceptive sensibility. The scale consists of eight subscales, as outlined in Table 3:

**Table 3:** Subscales of the MAIA.

Subscale	Description	Number of items
Noticing	How much an individual is aware of their bodily sensations such as breathing and heart rate	4
Not-distracting	The tendency not to ignore or distract oneself from sensations of pain or discomfort from the body	3
Not-worrying	The tendency not to experience emotional distress or worry with sensations of pain or discomfort from the body	3
Attention regulation	The ability to sustain and control attention to bodily sensations	7
Emotional awareness	The awareness of the connection between body signals and emotional states	5
Self-regulation	The ability to regulate psychological distress by attention to bodily sensations	4
Body listening	The tendency to actively listen to the body for insight	4
Trusting	The experience of one's body as safe and trustworthy	3

Responses are made on a 6-point Likert scale, in which participants indicate how often each statement applies to them generally in daily life, with responses from 0 (never) to 5 (always). The score for each subscale is calculated by the mean of its individual items, with no global score. The MAIA is found to have good convergent and discriminant validity and acceptable internal consistency^43^. Internal reliability is good for all subscales in both pregnant (α = 0.65–0.90) and non-pregnant (α = 0.66–0.91) samples.

#### The body understanding measure for pregnancy scale^7^ (BUMPs)

BUMPs is a 19 item self-report questionnaire designed to capture body satisfaction specifically in pregnant women. The scale consists of three subscales, as illustrated in Table 4.

**Table 4:** Subscales of the BUMPs.

Subscale	Description	Number of items
Appearance	Satisfaction with appearing pregnant	9
Weight	Capturing weight gain concerns	7
Physical	Relating to physical burdens of pregnancy	3

Scores are calculated by summing items for individual subscales and all 19 items for a global score. Higher scores indicate higher levels of dissatisfaction. The measure is suitable for women across all three trimesters, with good internal consistency across all scales (α = 0.71–0.91) and good test–retest reliability (0.78–0.93)^7^. The current sample is good for all scales (α = 0.74–0.90).

All participants completed the BCS and the MAIA, and the pregnant women additionally completed the BUMPs.

### Data analysis

Our hypotheses were tested using mixed ANCOVA and follow-up Bonferroni corrected independent t tests and Pearson’s correlations. Bayes factors are included for all follow-up t tests. To examine if interoception can predict both pregnancy specific and general body satisfaction we used hierarchical multiple regression. These analyses were also followed up with mediation analysis.

## Results

### Potential covariates

An ANOVA with the factors pregnancy (pregnant vs not pregnant) and having children (with children vs without children) was used to determine if age differed between groups. There was no main effect of pregnancy (F(1,494) = 0.19, p = 0.664, η_p_^2^ = 0.000, BF = 0.216) and no pregnancy ^*^ having children interaction (F(1,494) = 2.76, p = 0.097, η_p_^2^ = 0.006, BF = BF = 1.1e+14). However, the main effect of having children was significant (F(1,494) = 82.3, p < 0.001, η_p_^2^ = 0.143, BF = 1.76e+15) as those with children (mean = 34.3, SD = 4.22) were older than those without children (pregnant = 30.5, SD = 4.92). Therefore, age was used as a covariate in subsequent analyses.

### Effect of parity and gestation on pregnancy specific body satisfaction

First, to test our hypothesis that multiparous women would have lower body satisfaction compared to primiparous and that dissatisfaction with appearance and weight concerns would reduce with gestation, whilst frustrations with physical burdens would increase, a 2 × 3 mixed ANCOVA was conducted on BUMPs subscale scores. The between factor was parity (primiparous vs multiparous) and the within factor was BUMPs subscale (appearance, weight, physical). Due to the under-representation of women in the first trimester^7^ and differing ways to compute the stage of pregnancy in the literature [e.g.^31,54^], we decided to analyse gestational weeks as a continuous variable and enter it as a covariate along with age.

There was a main effect of BUMPs subscale (F(1,255) = 19.45, p < 0.001, η_p_^2^ = 0.071) although this was meaningless due to the inherent differences in subscale items and subsequent summed scores. There was also a significant main effect of parity (F(1,255) = 9.11, p = 0.003, η_p_^2^ = 0.034). Both the main effect of gestation (F(1,255) = 1.32, p = 0.252, η_p_^2^ = 0.005) and the main effect of age (F(1,255) = 3.0, p = 0.085, η_p_^2^ = 0.012) were non significant. The main effects were further described by significant interactions.

As anticipated, there was a significant interaction between BUMPs subscale and parity (F(2,510) = 4.34, p = 0.013, η_p_^2^ = 0.017). There was also a significant interaction between BUMPs subscale and gestation (F(2,510) = 3.59, p = 0.028, η_p_^2^ = 0.014). There was no significant interaction between BUMPs subscale and age (F(2,510) = 1.59, p = 0.205, η_p_^2^ = 0.006).

To follow up the BUMPs subscale ^*^  parity interaction and to determine which of the pregnancy body satisfaction constructs differed as a function of parity, three independent t tests were conducted correcting for multiple comparisons (critical alpha = 0.017). In line with our hypotheses, there was a significant effect of parity on BUMPs appearance subscale supported by the Bayes factor (t(257) =  − 2.96, p = 0.003, *d* =  − 0.37, BF = 8.21) with multiparous women having greater dissatisfaction (M = 26.7) than primiparous (M = 24.1). Contrary to predictions there was no effect of parity on BUMPs weight subscale (t(257) =  − 1.29, p = 0.198, *d* =  − 0.16, BF = 0.30) or BUMPS physical subscale (t(257) =  − 0.96, p = 0.338, *d* =  − 0.12, BF = 0.21). These results suggest that parity does influence pregnancy specific body satisfaction in terms of satisfaction with appearing pregnant, such that women in their first pregnancy have higher body satisfaction than women in subsequent pregnancies. See Table 5.

**Table 5:** Means (SD) of primiparous and multiparous women for the different body satisfaction measures.

Measure	Primiparous (N = 142)	Multiparous (N = 117)
BUMPs appearance	24.1 (7.2)	26.7 (6.9)
BUMPs weight	20.0 (6.2)	21.0 (6.4)
BUMPs physical	9.9 (3.1)	10.3 (3.1)
BCS	143.8 (22.8)	139.2 (20.5)

For the BCS higher values represent greater satisfaction, higher BUMPs values represent greater dissatisfaction.

*BCS* body cathexis scale, *BUMPs* body understanding measure for pregnancy scale.

To follow up the BUMPs subscale ^*^  gestation interaction, three Pearson’s correlations were conducted with a Bonferroni critical alpha of 0.017. There was a significant weak positive correlation between gestation and BUMPs physical subscale (r = 0.157, p = 0.011). No significant relationships were found between gestation and either BUMPS appearance subscale (r = 0.157, p = 0.011) or BUMPS weight subscale (r = 0.049, p = 0.434). These results suggest that gestation impacts pregnancy specific feelings towards the body in terms of the physical burdens of pregnancy, with which women become less satisfied the closer they get to birth.

### Effect of pregnancy and having children on body satisfaction

To examine our next hypothesis that previously having children would drive dissatisfaction with the body more than pregnancy a two-way ANCOVA was conducted on BCS scores. Independent variables were pregnancy (pregnant vs not pregnant) and having children (with children vs without children).

As anticipated, there was a significant main effect of having children, (F(1,498) = 14.67, p < 0.001, η_p_^2^ = 0.029, BF = 39.9) such that women who have had children reported lower body satisfaction (mean = 136, SD = 23.5) compared to women without children (mean = 143, SD = 23.1). There was also a significant effect of pregnancy (F(1,498) = 3.95, p = 0.047, η_p_^2^ = 0.008, BF = 1.23), with pregnant women having higher body satisfaction (mean = 142, SD = 21.8) than non-pregnant women (mean = 137, SD = 25.2). The interaction between pregnancy and having children was not significant (F(1,498) = 1.65, p = 0.20, η_p_^2^ = 0.003). The effect of age was also not significant (F(1,498) = 3.45, p = 0.064, η_p_^2^ = 0.007).

### The effect of parity and gestation on interoceptive sensibility

In order to examine our prediction that parity and gestation will impact interoceptive sensibility during pregnancy we conducted a 2 × 8 mixed ANCOVA. The between factor was parity (primigravida vs multigravida) and the within factor was MAIA subscale (noticing, not distracting, not worrying, attention regulation, emotional awareness, self regulation, body listening, body trusting). Gestation was entered as a covariate along with age. See Table 6 for descriptive data.

**Table 6:** Means (SD) of primiparous and multiparous women for the MAIA subscales.

Measure	Primiparous (N = 142)	Multiparous (N = 117)
MAIA noticing	3.4 (0.87)	3.2 (0.93)
MAIA not distracting	2.2 (0.94)	2.2 (0.93)
MAIA not worrying	2.5 (0.93)	2.7 (0.94)
MAIA attention regulation	2.7 (0.83)	2.6 (0.82)
MAIA emotion awareness	3.2 (0.92)	3.2 (0.98)
MAIA self regulation	2.7 (0.97)	2.5 (0.94)
MAIA body listening	2.2 (1.1)	1.9 (0.99)
MAIA body trusting	3.2 (1.1)	2.8 (1.1)

Higher scores indicate higher levels of awareness.

*MAIA* multidimensional assessment of interoceptive awareness.

There was a significant main effect of parity (F(1,255) = 5.74, p = 0.017, ηp^2^ = 0.02) and age (F(1,255) = 9.272, p = 0.003, ηp^2^ = 0.035). There was no significant main effect of MAIA subscale (F(7,1785) = 1.58, p = 0.003, ηp^2^ = 0.006) or gestation (F(1,255) = 0.221, p = 0.638, ηp^2^ = 0.001).These main effects were better described by the interactions below.

There was a significant MAIA subscale ^*^ parity interaction (F(7, 1785) = 2.72, p = 0.008, η_p_^2^ = 0.011). There was also a significant interaction between MAIA subscale and gestation (F(7, 1785) = 2.87, p = 0.006, η_p_^2^ = 0.011). There was no significant MAIA subscale ^*^ age interaction (F(7, 1785) = 0.56, p = 0.788, η_p_^2^ = 0.002).

To follow up the MAIA subscale ^*^ parity interaction eight independent t tests corrected for multiple comparisons (critical alpha = 0.0063) were conducted. As anticipated there was a significant effect of parity on body trusting (t(257) = 2.89, p = 0.004, *d* = 0.36, BF = 6.78). with primiparous women having greater body trusting (mean = 4.23, SD = 1.06) compared to multiparous (mean = 3.84,SD = 1.09).

Contrary to predictions, there were no significant effects of parity on noticing (t(257) =  − 2.02, p = 0.04, *d* = 0.25, BF = 0.94), not-distracting (t(257) =  − 0.52, p = 0.604, *d* = 0.065, BF = 0.16), not-worrying (t(257) =  − 1.25, p = 0.213, *d* = 0.15, BF = 0.29), attention regulation (t(257) = 0.066, p = 0.507, *d* = 0.08, BF = 0.17), emotional awareness (t(257) =  − 0.173, p = 0.863, *d* = 0.02, BF = 0.14), self regulation (t(257) = 1.78, p = 0.08, *d* = 0.22, BF = 0.61) or body listening (t(257) = 1.59, p = 0.112, *d* = 0.20, BF = 0.46) - see Supplementary Material S1.

To follow up the MAIA subscale ^*^ gestation interaction eight Pearson’s correlations were conducted with a bonferroni critical alpha of 0.0063. Contrary to predictions, none of the relationships between the MAIA subscales and gestation survived corrections for multiple comparisons: noticing (r = 0.158, p = 0.011), not distracting (r =  − 0.138, p = 0.026), not worrying (r =  − 0.015, p = 0.814), attention regulation (r =  − 0.05, p = 0.42), emotional awareness (r = 0.09, p = 0.170), self regulation (r = 0.137, p = 0.027), body listening (r = 0.076, p = 0.223) and body trusting (r = 0.005, p = 0.932). See Table 6 for descriptive data.

### The effect of pregnancy and having children on interoceptive sensibility

To specifically examine if interoceptive sensibility is influenced by being pregnant or already having children we conducted a 2 × 2 × 8 ANCOVA controlling for age as a covariate. The between factors were pregnancy (pregnant vs not pregnant) and having children (with children vs without children). The within factor was MAIA subscale (noticing, not distracting, not worrying, attention regulation, emotional awareness, self regulation, body listening and body trusting).

There was a significant main effect of MAIA subscale (F(7,3465) = 5.93, p < 0.001, ηp^2^ = 0.001), having children (F(1,495) = 10.20, p = 0.017, ηp^2^ = 0.02) and age (F(1,495) = 5.34, p = 0.021, ηp^2^ = 0.011). There was no significant main effect of pregnancy (F(1,495) = 1.01, p = 0.314, ηp^2^ = 0.002) (see supplementary material S2).

There was no significant interaction between pregnancy and having children (F(1,495) = 0.217, p = 0.642, ηp^2^ < 0.001), MAIA subscale and age (F(7,3465) = 0.85, p = 0.544, ηp^2^ = 0.002) or MAIA subscale, pregnancy and having children (F(7,3465) = 3.22, p = 0.002, ηp^2^ = 0.006). There was a significant interaction between MAIA subscale and pregnancy (F(7,3465) = 3.22, p = 0.002, ηp^2^ = 0.006), and between MAIA subscale and having children (F(7,3465) = 3.22, p = 0.002, ηp^2^ = 0.006).

To follow up the MAIA subscale ^*^ pregnancy interaction eight independent t tests corrected for multiple comparisons (critical alpha = 0.0063). These analyses allowed us to directly examine the hypothesis that interoceptive sensibility would be different in pregnant women compared to non-pregnant women. As expected, there was a significant effect of pregnancy on not distracting (t(498) =  − 2.99, p = 0.003, *d* =  − 0.27, BF = 7.42). Pregnant women reported higher scores (mean = 2.2, SD = 0.93) compared to non-pregnant women (mean = 1.9, SD = 0.96), suggesting that pregnant women avoid signals less compared to non-pregnant women.

Contrary to predictions, there were no significant effects of pregnancy on noticing (t(498) =  − 2.33, p = 0.02, *d* = 0.21, BF = 1.37), not worrying (t(498) =  − 0.969, p = 0.333, *d* =  − 0.09, BF = 0.16), attention regulation (t(498) =  − 1.27, p = 0.204, *d* = 0.11, BF = 0.22), emotional awareness (t(498) = 1.16, p = 0.247, *d* = 0.10, BF = 0.19), self regulation (t(498) =  − 1.75, p = 0.08, *d* =  − 0.16, BF = 0.44), body listening (t(498) =  − 0.70, p = 0.488, *d* =  − 0.062, BF = 0.46) or body trusting (t(498) =  − 1.11, p = 0.269, *d* =  − 0.10, BF = 0.18).

To follow up the MAIA subscale × having children interaction eight independent t tests corrected for multiple comparisons (critical alpha = 0.0063). These analyses allowed us to directly examine the hypothesis that interoceptive sensibility would be different in women with children compared to women without children.

As anticipated, there was a significant effect of having children on body trusting (t(498) =  − 3.98, p < 0.001, *d* = 0.36, BF = 189.8). Those without children reported greater body trusting (mean = 4.23, SD = 1.06) compared to those with children (mean = 3.84, SD = 1.09).

Contrary to predictions, there were no significant effects of having children on noticing (t(498) =  − 2.20, p = 0.028, *d* = 0.20, BF = 1.04), not distracting (t(498) =  − 1.8, p = 0.073, *d* = 0.16, BF = 0.48), not worrying (t(498) = 0.251, p = 0.802, *d* = 0.02, BF = 0.10), attention regulation (t(498) =  − 0.405, p = 0.685, *d* =  − 0.04, BF = 0.11), emotional awareness (t(498) = 0.419, p = 0.675, *d* = 0.04, BF = 0.11), self regulation (t(257) =  − 1.72, p = 0.086, *d* = 0.15, BF = 0.42) or body listening (t(498) =  − 1.79, p = 0.075, *d* = 0.16, BF = 0.47). See Table 7 for descriptive data.

**Table 7:** Means (SD) of pregnant and non-pregnant women and those with and without children for the MAIA subscales.

Measure	Pregnant (N = 259)	Not pregnant (N = 241)	Children (N = 250)	No children (N = 250)
MAIA noticing	3.3 (0.91)	3.5 (0.86)	3.3 (0.93)	3.5 (0.81)
MAIA not distracting	2.2 (0.93)	2.0 (0.96)	2.0 (0.98)	2.1 (0.93)
MAIA not worrying	2.6 (0.94)	2.5 (0.96)	2.6 (0.97)	2.5 (0.93)
MAIA attention regulation	2.7 (0.83)	2.6 (0.87)	2.6 (0.85)	2.7 (0.85)
MAIA emotion awareness	3.2 (0.95)	3.3 (1.0)	3.3 (1.0)	3.2 (0.94)
MAIA self regulation	2.7 (0.96)	2.5 (0.95)	2.5 (0.95)	2.7 (0.96)
MAIA body listening	2.1 (1.1)	2.0 (1.1)	1.9 (1.1)	2.1 (1.1)
MAIA body trusting	3.1 (1.1)	3.0 (1.2)	2.8 (1.2)	3.2 (1.2)

Higher scores indicate higher levels of awareness.

*MAIA* multidimensional assessment of interoceptive awareness.

### Predictors of pregnancy body satisfaction

To examine predictions that constructs of interoceptive sensibility, particularly body trusting, would be significant predictors of feelings toward pregnancy specific body changes, we ran a forced entry hierarchical multiple regression on our pregnant participants with BUMPs total score as the outcome variable (shown in Table 8).

**Table 8:** Summary of hierarchical regression analysis predicting pregnancy specific body satisfaction.

Model	Outcome variable	Predictor	β	R^2^	Adjusted R^2^	F	Δ R^2^	ΔF
Step 1	BUMPs	Age	− 0.05	0.002	− 0.002	0.574		
Step 2	BUMPs	Age	− 0.11	0.037	0.026	3.30**	0.035	3.52**
		Parity	0.20**					
		Gestation	0.025					
Step 3	BUMPs	Age	− 0.03	0.515	0.232	8.84***	0.21	11.75***
		Parity	0.10					
		Gestation	0.03					
		Noticing	0.10					
		Not distracting	0.09					
		Not worrying	0.02					
		Self-regulation	− 0.11					
		Attention regulation	0.03					
		Emotion awareness	0.05					
		Body listening	0.025					
		Body trusting	− 0.47***					

*BUMPs* body understanding measure in pregnancy scale.

*p < 0.5, **p < 0.01, ***p < 0.001.

In stage one we entered age as a possible predictor. The model was not significant (F(1,257) = 0.574, p = 0.449). In stage two we entered gestation and parity, the model was significant (F(3,255) = 3.30, p = 0.021) accounting for 2.6% or the variance. Whether or not women were in their first pregnancy was the only significant predictor (t(3,255) = 3.02, p = 0.003). In stage three we added MAIA subscales: noticing, not distracting, not worrying, attention regulation, emotion awareness, self regulation, body listening and body trusting. The model was significant (F(11,247) = 8.08, p < 0.001) accounting for 23% of the variance. The only significant predictor was body trusting (t(11,247) = − 7.03, p < 0.001). Whether or not the women were in their first pregnancy was no longer significant.

### Mediation analysis for body trusting on the relationship between parity and pregnancy body satisfaction

**Figure 1:**
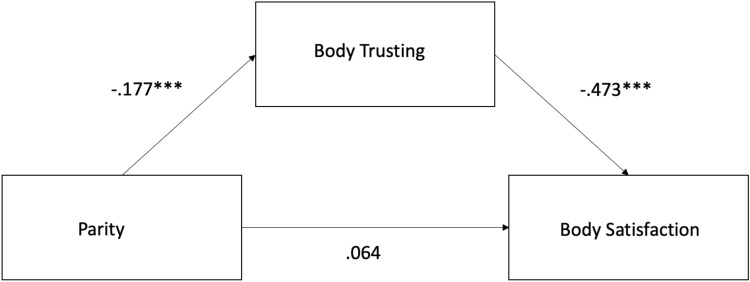
Mediation results for the relationship between parity and pregnancy body satisfaction. Body trusting was found to be a significant mediator, such that differences in body satisfaction between primiparous and multiparous women are mediated by changes in body trust.

To follow up the significant effect of the body trusting subscale on pregnancy specific body satisfaction (BUMPS) we conducted a mediation analysis, with parity as the independent variable, BUMPs as the outcome variable and body trusting as a potential mediator. The mediation analysis was conducted in Rstudio(*lavaan package*). The regression coefficients between parity and body trusting (β =  − 0.177, CI =  − 0.569 to − 0.377, p = 0.004) and between body trusting and pregnancy body satisfaction (BUMPS) were significant (β =  − 0.473, CI =  − 0.294 to − 0.060, p < 0.001) as well as the indirect effect (β = 0.084, CI = 0.026 to 0.142, p = 0.006). The regression coefficient between parity and body satisfaction (BUMPs) was not significant (β = 0.064, CI =  − 0.044—0.171, p = 0.248). See Fig. 1. The results indicate that the effect of Parity on pregnancy body satisfaction was fully mediated by body trusting.

### Predictors of general body satisfaction

To examine predictions that constructs of interoceptive sensibility, particularly body trusting, would be significant predictors of body satisfaction across the entire sample, we ran a forced entry hierarchical multiple regression with BCS as the outcome variable (see Table 9).

**Table 9:** Summary of hierarchical regression analysis predicting body satisfaction.

Model	Outcome variable	Predictor	β	R^2^	Adjusted R^2^	F	Δ R^2^	ΔF
Step 1	BCS	Age	0.008	7.70e−5	− 0.002	0.038		
Step 2	BCS	Age	0.083	0.039	0.033	6.71***	0.039	10.0***
		Pregnancy	0.18					
		Children	0.36***					
Step 3	BCS	Age	0.013	0.394	0.381	28.89***	0.355	35.8***
		Pregnancy	0.1081					
		Children	0.127					
		Noticing	− 0.126**					
		Not distracting	− 0.106**					
		Not worrying	− 0.035					
		Self-regulation	0.036					
		Attention regulation	− 0.002					
		Emotion awareness	0.042					
		Body listening	− 0.003					
		Body trusting	− 0.562***					

*BCS* body cathexis scale.

*p < 0.5, **p < 0.01, ***p < 0.001.

In stage one we entered age as a possible predictor. The model was non-significant (F(1,501) = 0.039, p = 0.844). In stage two we entered whether or not the women were pregnant and whether or not they had children already. The model was significant, accounting for 2.8% of the variance (F(3,499) = 5.84, p < 0.001). The only significant predictor was having children (t(3,499) = 3.77, p < 0.001). In the third stage we added MAIA subscales: noticing, not distracting, not worrying, attention regulation, emotion awareness, self regulation, body listening and body trusting The model was significant (F(11,491) = 26.12, p < 0.001) accounting for 35% of the variance. Significant predictors were noticing (t(11,491) =  − 2.94, p = 0.003), not distracting (t(11,491) = 2.02, p = 0.044) and body trusting (t(11,491) = 13.73, p < 0.001). Whether or not the women had children was no longer a significant predictor.

### Mediation analysis for body trusting on the relationship between having children and body satisfaction

**Figure 2:**
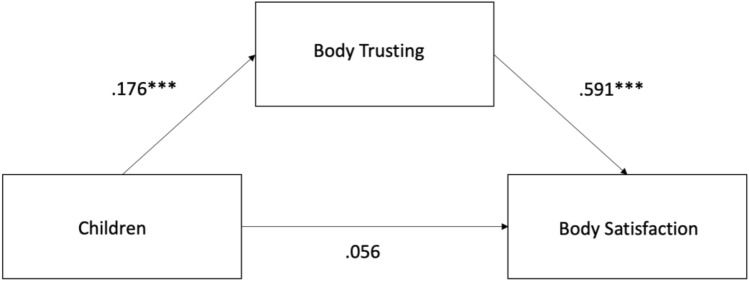
Mediation effect of body trusting on the relationship between having children and body satisfaction. Body trusting was found to be a significant mediator, such that differences in body satisfaction between those with and without children are mediated by changes in body trust.

To follow up the significant effect of the body trusting subscale on body satisfaction we conducted a mediation analysis, with having children (with vs. without children) as the independent variable, BUMPs as the outcome variable and body trusting as a potential mediator. The mediation analysis was conducted in Rstudio(*lavaan package*). The regression coefficients between children and body trusting (β = 0.176, CI = 0.091–0.260, p < 0.001) and between body trusting and body satisfaction (BCS) were significant (β = 0.591, CI = 0.533–0.649, p < 0.001) as well as the indirect effect (β = 104, CI = 0.053–0.155, p < 0.001). The regression coefficient between children and body satisfaction (BSC) was not significant (β = 0.056, CI =  − 0.015 to 0.126, p = 0.125). The results suggest that the effect of having children on body satisfaction is fully mediated by body trusting, see Fig. 2.

## Discussion

The current results show a significant effect of already having children on women’s body satisfaction in both pregnant and non-pregnant samples. Thus, although pregnant women reported less body dissatisfaction than non-pregnant women, this effect was driven more by parental status with primiparous women and non-pregnant women without children being more satisfied with their body compared to those who were already mothers. Furthermore, these effects were mediated by levels of trusting in the body (measure of interoceptive sensibility). Body trusting was found to be reduced in women with children and was the strongest predictor of both general and pregnancy specific body satisfaction. Additionally, there was a difference in interoceptive avoidance between pregnant and non-pregnant women, such that pregnant women distracted less from feelings of pain and discomfort compared to non-pregnant women. During pregnancy there was a significant effect of gestation on feelings towards the physical burdens of pregnancy, with women feeling more frustrated/negative about pregnancy related physical constraints the closer they got to birth. None of the other constructs of interoceptive sensibility and body satisfaction were significantly different between pregnant and non-pregnant samples or with those who have children. Age had no significant effects on individual constructs of body satisfaction or interoceptive sensibility.

There are mixed findings in the literature regarding body satisfaction during pregnancy, with some studies suggesting an improvement^31,55,56^, some a worsening^57,58^ and others suggesting relative stability in body satisfaction^59^. It has been proposed that these mixed findings represent a heterogeneous experience during the perinatal period^7^, which is often reflected in qualitative studies (e.g.^60^). Here, we found that pregnant women were less dissatisfied with their body than non-pregnant women. However, we also suggest that the parental status of both the pregnant and the comparison group may be an additional contributing factor to the prior (seemingly contradictory) results. Rather than body satisfaction being specifically related to pregnancy, it seems that whether or not women already have children is a stronger factor influencing how women feel about their body, with women who have had children reporting lower body satisfaction than women without children. Such decreases in body satisfaction may be due to permanent changes to the body as a result of pregnancy^39,40^, changes in social status, such as motherhood not being associated with attractiveness^61^, as well as changes in self identity and social role^61,62^. Furthermore, women with children also have less time to meet their own needs, at least when the child(ren) is young^63^, resulting in women having less time to focus on their own appearance and thus negatively impacting body satisfaction. This suggests that findings of relative differences in body satisfaction would depend, at least in part, on parity status of a pregnant sample as well as parental status of a control sample.

Although both the social and physical factors are likely to play a role in how mothers feel about their body, the significant role of body trusting on levels of body satisfaction may suggest that physical aspects are more important. Indeed, for both general and pregnancy specific body satisfaction, the relationship between feelings towards the body and having had children was fully mediated by body trusting. This means that the mechanism through which women with children (pregnant or not) have lower body satisfaction may be via changes (worsening) in body trusting. One reason for a reduction in body trusting could be physical consequences of previous pregnancy and birth. Many women experience permanent physical changes as a result of previous pregnancy and birth, which may impact the way in which the body functions^40^ and thus undermine trust. The act of birth itself may also influence body trust. Health practitioners’ observations of signs of birth trauma suggest that trauma in relation to birth could impact over a third of new mothers, with ongoing implications on wellbeing^64^. Moreover, recent qualitative research found that as much as 65% of new mothers feel some sort of failure in relation to birth^65^. The authors suggested that feelings of self-perceived failure surrounding birth are driven by self-imposed and societal expectations. For many women deviation from what is perceived as an ideal ‘natural birth’, such as involvement of medical interventions, can be seen as failing to birth ‘properly’ and even failing their baby. Such medical interventions can range from emergency c-sections to following medical protocols or taking pain medication. Some of the participants reported that agreeing to such medical interventions was felt as a personal weakness, that they were not tough enough or lacked the mental and/or physical strength to birth naturally but also that their body didn’t work properly and was out of their control. Given that the act of giving birth is so physical, feelings of failure or trauma from birth may serve to undermine women’s trust in their body and thus may make women who have children more vulnerable to negative feelings about their body. Although these interpretations are speculative, these current results may have important implications on how women are supported through their transition to parenthood to assist their ongoing wellbeing and facilitate body trust and thus body satisfaction. Whether or not the reported lower body satisfaction and body trusting in women with children is driven by physical or social changes, or a combination of these, we suggest that such factors need to be considered when examining differences in bodily experience before, during and after pregnancy.

Somewhat surprisingly, pregnant women demonstrated equivalent worry about their bodily signals compared to non-pregnant women. Intuitively it might seem that pregnant women would show more worry when attending to their body because of the additional concern regarding the wellbeing of the fetus. However, when considering the nature of the questions in the not worrying subscale of the MAIA, the focus is on pain and discomfort, for example, “I can notice an unpleasant body sensation without worrying about it”. Pain is a common and intrinsic part of pregnancy; it has been suggested that the prevalence of lower back and pelvic pain in pregnant women is over 70%^66,67^. Therefore, the sort of discomfort captured by the MAIA may be anticipated during pregnancy and thus may not lead to additional worry. On the other hand, the experience of worry may relate to an intuitive link to the wellbeing of the fetus, but demonstrate a heterogeneous experience across women, similar to that observed with body satisfaction. Therefore, considering the individual experience, or longitudinal changes may be more important rather than studying global differences between pregnant and not pregnant samples.

Pregnant women reported reduced distracting from bodily signals (higher scores on the not distracting subscale) compared to non-pregnant women. This suggests that women avoid feelings of pain and discomfort less when they are pregnant. This may be due to health concerns for the fetus, wanting to be aware of early signs of labour or just being allowed to feel uncomfortable during pregnancy due to the clear visual marker of discomfort (baby bump) and a social expectation of discomfort during pregnancy. Contrary to our predictions, we did not find a significant effect of pregnancy or gestation on body listening. It was anticipated that pregnant women may listen to their body more for signals relating to the fetus, such as fetal movement, hunger and even signs of labour later on in pregnancy. A previous longitudinal study did find increased listening during later compared to early pregnancy and compared to the postpartum^44^. Although the listening subscale of the MAIA includes an item which may be very relevant to pregnancy and labour/birth “I listen to my body to inform me about what to do”, the other two items focus on attention to the body in relation to emotions; “I listen to information from my body about my emotional state” and “When I am upset, I take time to explore how my body feels”. Therefore, the MAIA may not be able to capture specific changes in how we attend to our bodies during pregnancy and the way in which pregnant women respond to these items may not be equivalent to responses outside of the perinatal period. Similarly we did not find a significant effect of noticing and indeed, for the majority of our predictions involving the MAIA subscales, we did not find significant differences in interoceptive sensibility between pregnant and non-pregnant samples as well as finding no evidence for changes relative to gestational age. This may be that the scale does not capture relevant constructs or it may well reflect a stability of subjective interoceptive experience throughout the perinatal period, with individual differences in the measures being potentially more important for wellbeing.

We also found evidence for differences in frustrations with physical burdens of pregnancy across gestation. Previous studies have also reported that different aspects of our bodily experience change in different ways during the course of pregnancy. For example, it has been found that during the final trimester of pregnancy, women felt less fat compared to at any other time in pregnancy and their retrospective account prior to pregnancy^34^, whereas other research suggests that body image worsens in the third trimester^58^. The current results suggest that women later in pregnancy have more negative feelings about physical burdens of pregnancy. Together, these results suggest that the point during pregnancy at which measures were taken could also affect outcomes of the measurements.

When considering the current findings we have to do so in the context of the study limitations. Firstly, it should be noted that as this was a cross-sectional study, changes in interoceptive sensibility can only be inferred. Therefore, although we find evidence for differences between groups and throughout gestation, to assess whether these experiences change over the perinatal period our findings should be further examined in longitudinal studies. Additionally, a main factor examined by this study is comparing women with and without children with the assumption that those with children had undergone the experience of pregnancy and childbirth. The survey asked participants how many children they had, not whether they had previously been pregnant before or if they had given birth, which in some circumstances could be ambiguous for example for women who have adopted, or used surrogacy. Likewise having been pregnant before does not necessarily mean the same as having children, due to adoption, miscarriage or stillbirth. Future work examining these questions in women who have children, but without ever giving birth and women who have been pregnant, but without raising children, may help tease apart the relative roles of social and physical factors to interoceptive sensibility and body satisfaction. The current sample is also compromised by a lack of inclusiveness, such that the sample consists predominantly of white, middle class, cis-gender women. We know that culture, ethnicity, gender and socioeconomic status can be important for social norms, body satisfaction and even maternity care^68–71^, therefore these aspects should be considered in future research.

Another limitation with the current study is the absence of Body Mass Index (BMI) data. BMI and body size are thought to relate to both body satisfaction^70,72^ and interoception^73–75^, particularly with women^76^ and in relation to high BMI and obesity^77^. A key physical symptom during pregnancy is weight gain therefore such measures as pre-pregnancy BMI and pregnancy related weight gain may play a key role in interoceptive experience during the perinatal period as well as body satisfaction. Future studies should aim to capture this information to specifically examine how it relates to the link between body trusting and body satisfaction. This may be particularly important relating to body trusting and weight gain. A previous study found that the weight subscale of BUMPs correlated with body trusting, which was interpreted as a greater trust in the body would mean that women would trust any weight gain to be essential for the healthy development of the fetus^7^. However, this relationship is likely to be moderated by actual weight gain, such that excessive weight gain in pregnancy may compromise body trust. Thus, future research should specifically examine this relationship.

Finally, it is important to note that some of the effect sizes of the current results are relatively small. Even though our key findings are supported by Baysian statistics, thus suggesting they are unlikely to be false positives, it has been suggested that small effect sizes do not provide information that is relevant to everyday thoughts and feelings^78^, which may draw into question how meaningful some of the above mentioned effects are. However, more recently it has been suggested that even small effect sizes can be valuable especially in complex fields such as mental health, including body (dis)satisfaction, and the effect sizes found were comparable with other meaningful texts related to bodily experience in pregnancy (e.g.^79^). With such complex phenomena it is unlikely that single constructs or mechanisms underlie individual differences, and even small effects can have a big impact on those who are already vulnerable^80^. Additionally, due to the fact that the MAIA is not developed for, or validated in, pregnant women, we may also be missing important interoceptive changes, which are not captured by this measure, and thus underestimating interoceptive changes at this time. Much of the MAIA scale focuses on pain and discomfort, and whilst this is relevant in pregnancy, other aspects of interoception, such as hunger and thirst, may be attended to differently during pregnancy and may also have implications on body satisfaction through weight gain. Likewise it is important to consider which elements of body satisfaction measures such as the BCS are capturing. This is particularly the case during pregnancy, given that the BCS asks specifically about satisfaction with certain body parts. It has been proposed that there is a change in emphasis concerning the body during pregnancy^60^, it is therefore conceivable that pregnant women are responding more in terms of satisfaction with the function of certain body areas, whereas non-pregnant women may respond more in terms of appearance and so we may not be comparing equivalent constructs between the groups. Another potential issue to consider is in relation to current definitions of what sensations interoception refers to. Many of the MAIA subscales refer to pain and discomfort: whilst all types of pain are included in recent liberal definitions of interoception^81^, it is conceivable that these sensations may derive from an exteroceptive source. Accepted definitions of interoception have moved from very restrictive, only involving the viscera, to more inclusive criteria, including all sensations which follow specific ‘interoceptive’ neural pathways^11,81^. Such inclusive definitions not only include all types of pain, but also certain types of touch^81,82^. Scales, such as the MAIA focus predominantly on these more liberal constructs of interoception opposed to traditional elements like hunger and thirst and as such may be missing key elements of the interoceptive experience, particularly during pregnancy. Moreover, particularly when considering interoceptive sensibility, broad interoception definitions may be even more difficult to truly capture, given potential individual differences in the subjective experience of their body as well as item interpretation (i.e. we do not know what sort of pain they are referring to). This is also impeded by the observations that interoceptive sensibility measures like the MAIA do not tend to correlate with more objective measures of interoception^83^ and this also seems to be the case during pregnancy^22^, which means these measures are difficult to fully validate. However, despite these shortcomings, the MAIA is an accepted and well used measure of interoceptive sensibility. Future work should specifically aim to examine interoceptive experience in pregnancy and the suitability of scales like the MAIA to capture such constructs.

Despite these limitations, our current results suggest that body satisfaction is reduced in women who already have children, with a higher impact than pregnancy status, thus parental status should be examined in studies assessing level and impact of body satisfaction in pregnant and non-pregnant samples. Importantly, this relationship between having had children and body satisfaction is mediated by body trusting and thus highlights a key area of future research for body satisfaction interventions. Avoidance of interoceptive signals was also found to vary during pregnancy. However, existing measures are limited in scope of interoceptive signals, which may not be relevant to pregnancy and thus may be underestimating interoceptive changes during the perinatal period.

## Supplementary Information

Supplementary Information.

## Data Availability

The datasets generated during and/or analysed during the current study are available from the corresponding author on reasonable request.
