# Supplementary material for: Interoceptive sensibility and body satisfaction in pregnant and non-pregnant women with and without children
Source: Sci Rep. 2022 Sep 27;12:16138. doi: 10.1038/s41598-022-20181-z (PMC9515153; doi:10.1038/s41598-022-20181-z)
Supplement: Supplementary file 1 — Supplementary Information. [file 41598_2022_20181_MOESM1_ESM.pdf]

Supplementary material

S1:

Interoceptive sensibility during pregnancy

The ANCOVA conducted for the Not Worrying subscale revealed no significant effect of Parity ($F(1,256) =.15$, $p = .22$, $\eta_p^2 = .006$, BF = .29) and no significant effect of Gestation ($F(1,256) =.003$, $p = .96$, $\eta_p^2 < .001$, BF = .14).

The ANCOVA conducted for the Attention Regulation subscale revealed no significant effect of Parity ($F(1,256) =.77$, $p = .38$, $\eta_p^2 = .003$, BF = .17) and no significant effect of Gestation ($F(1,256) =.97$, $p = .32$, $\eta_p^2 = .004$, BF = .19).

The ANCOVA conducted for the Emotional awareness subscale revealed no significant effect of Parity ($F(1,256) =.26$, $p = .61$, $\eta_p^2 = .001$, BF = .14) and no significant effect of Gestation ($F(1,256) =2.12$, $p = .15$, $\eta_p^2 = .008$, BF = .33).

The ANCOVA conducted for the Body Listening subscale revealed no significant effect of Parity ($F(1,256) =1.81$, $p = .18$, $\eta_p^2 = .007$, BF = .46) and no significant effect of Gestation ($F(1,256) =.77$, $p = .38$, $\eta_p^2 = .003$, BF = .28).

The ANCOVA conducted for the Self Regulation subscale revealed the effect of Gestation approached significance ($F(1,256) = 3.49$, $p = .063$, $\eta_p^2 = .013$, BF = 1.4).

S2:

The effect of pregnancy and having children on interoceptive sensibility

The ANCOVA conducted for the Not Worrying subscale revealed no significant effect of Pregnancy ($F(1,496) = .98$, $p = .32$, $\eta_p^2 = .002$, $BF = .15$) and no significant effect of Children ($F(1,496) = .08$, $p = .77$, $\eta_p^2 < .001$, $BF = .10$).

The ANCOVA conducted for the Attention Regulation subscale revealed no significant effect of Pregnancy ($F(1,496) = 1.54$, $p = .22$, $\eta_p^2 = .003$, $BF = .22$) and no significant effect of Children ($F(1,496) = .065$, $p = .8$, $\eta_p^2 < .001$, $BF = .11$).

The ANCOVA conducted for the Emotional awareness subscale revealed no significant effect of Pregnancy ($F(1,496) = 1.26$, $p = .26$, $\eta_p^2 = .003$, $BF = .19$) and no significant effect of Children ($F(1,496) = .094$, $p = .8$, $\eta_p^2 < .001$, $BF = .11$).

The ANCOVA conducted for the Self Regulation subscale revealed no significant effect of Pregnancy ($F(1,496) = 2.53$, $p = .11$, $\eta_p^2 = .005$, $BF = .43$) and no significant effect of Children ($F(1,496) = 2.3$, $p = .13$, $\eta_p^2 = .005$, $BF = .42$).

The ANCOVA conducted for the Body Listening subscale revealed no significant effect of Pregnancy ($F(1,496) = .27$, $p = .60$, $\eta_p^2 < .001$, $BF = .13$). The effect of Children approached significance ($F(1,496) = 2.9$, $p = .09$, $\eta_p^2 = .006$, $BF = .46$).
